# Supplementary material for: Eye Movement Desensitization and Reprocessing versus Cognitive Behavior Therapy for Treating Post-Traumatic Stress Disorder: A Systematic Review and Meta-Analysis
Source: Int J Environ Res Public Health. 2022 Dec 15;19(24):16836. doi: 10.3390/ijerph192416836 (PMC9778888; doi:10.3390/ijerph192416836)

Table S1: Critical Appraisal for RCTs

Authors/Year of Publication	Random sequence generation adequate?	Allocation concealment adequate?	Blinding of participants adequate?	Blinding of personnel adequate?	Blinding of outcome assessors adequate?	Was incomplete outcome data addressed?	Was it free of selective reporting?
de Roos et al. (2011)	-	?	-	?	+	+	+
Nijdam et al. (2012)	+	+	-	?	+	+	+
Diehle et al. (2015)	+	+	-	-	+	+	+
van den Berg et al. (2015)	+	?	-	+	+	+	+
De Bont et al. (2016)	?	-	-	+	+	+	+
de Roos et al. (2017)	+	+	-	+	+	+	+
Nijdam et al. (2018)	+	-	-	-	+	+	+
Stanbury et al (2020)	+	+	-	+	+	+	+

+	Low Risk of bias	?	Unclear Risk of bias	-	High Risk of bias

Figure S1: Meta-analysis of the effects of post-treatment on PTSD symptoms

Study or Subgroup	EMDR Mean	EMDR SD	EMDR Total	CBT Mean	CBT SD	CBT Total	Weight	Std. Mean Difference IV, Random, 95% CI
de Roose 2011	12	9.1	26	12.3	8.1	26	13.4%	-0.03 [-0.58, 0.51]
de Roose 2017	51.03	19.23	43	56.63	22.05	42	15.4%	-0.27 [-0.70, 0.16]
Diehle 2015	23.6	30	25	22.1	23.3	23	13.1%	0.05 [-0.51, 0.62]
Nijdam 2012	19.94	11.81	70	31.11	12.47	70	16.7%	-0.91 [-1.26, -0.57]
Nijdam 2018	54.93	23.77	57	53.77	22.14	59	16.5%	0.05 [-0.31, 0.41]
Stanbury 2020	22.57	21.68	10	17.13	16.23	10	8.7%	0.27 [-0.61, 1.15]
van den Berg 2015	72.1	17.6	55	69.6	14.9	53	16.2%	0.15 [-0.23, 0.53]
**Total (95% CI)**			**286**			**283**	**100.0%**	**-0.14 [-0.48, 0.21]**

Heterogeneity: Tau² = 0.15; Chi² = 23.62, df = 6 (P = 0.0006); I² = 75%

Test for overall effect: Z = 0.77 (P = 0.44)

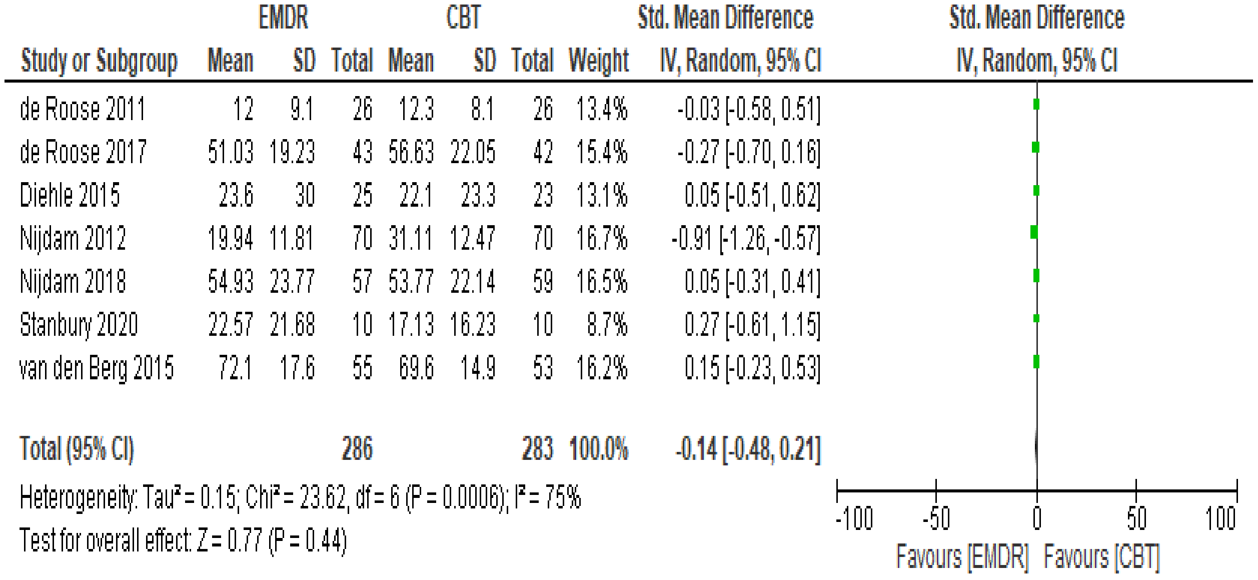

Figure S2: Meta-analysis of the effects of three-month follow-up on PTSD symptoms

	EMDR			CBT				Mean Difference
Study or Subgroup	Mean	SD	Total	Mean	SD	Total	Weight	IV, Fixed, 95% CI
de Roose 2011	11.2	8	26	11.9	8.3	26	64.1%	-0.70 [-5.13, 3.73]
de Roose 2017	35.58	14.07	51	37.36	15.34	45	35.9%	-1.78 [-7.70, 4.14]
Total (95% CI)			77			71	100.0%	-1.09 [-4.63, 2.46]

Heterogeneity: Chi² = 0.08, df = 1 (P = 0.77); I² = 0%

Test for overall effect: Z = 0.60 (P = 0.55)

Mean Difference
IV, Fixed, 95% CI

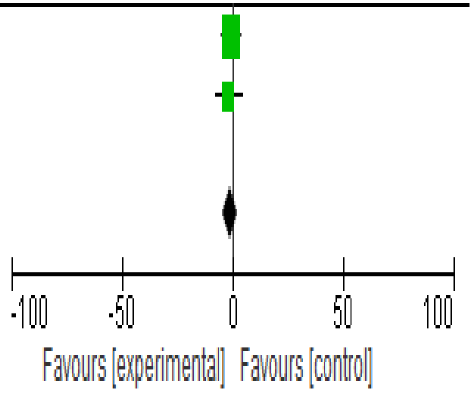

Figure S3: Meta-analysis of the effects of post-treatment on depression symptoms

	EMDR			CBT				Mean Difference
Study or Subgroup	Mean	SD	Total	Mean	SD	Total	Weight	IV, Fixed, 95% CI
De Bont 2016	22.2	44	55	18.3	44	53	0.8%	3.90 [-12.70, 20.50]
Diehle 2015	3.1	2	25	7.1	6	23	33.6%	-4.00 [-6.57, -1.43]
Nijdam 2012	5.67	4.54	70	7.38	6.42	70	65.6%	-1.71 [-3.55, 0.13]
**Total (95% CI)**			**150**			**146**	**100.0%**	**-2.43 [-3.93, -0.94]**

Heterogeneity: Chi² = 2.57, df = 2 (P = 0.28); I² = 22%

Test for overall effect: Z = 3.20 (P = 0.001)

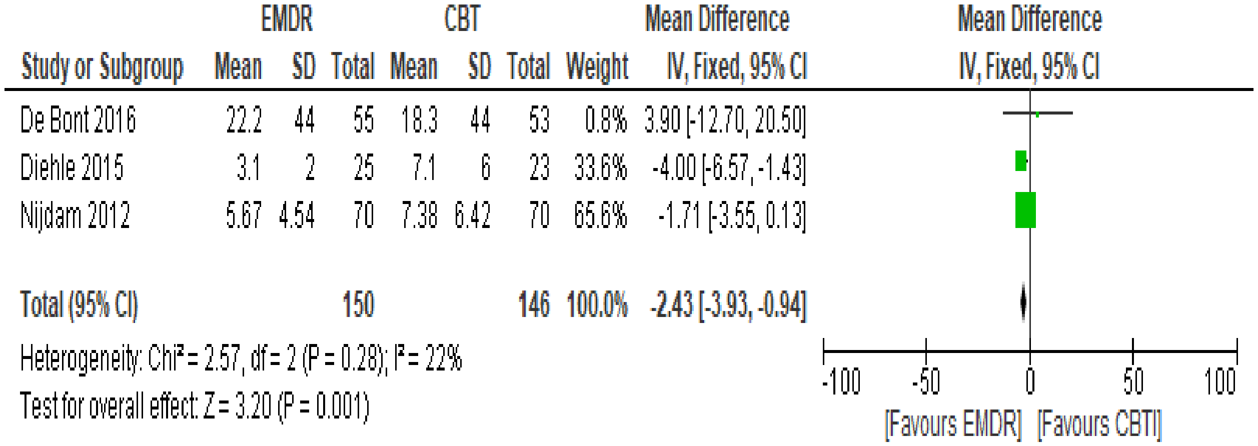

Figure S4: Meta-analysis of the effects of six-month follow-up on depression symptoms

	EMDR			CBT				Mean Difference
Study or Subgroup	Mean	SD	Total	Mean	SD	Total	Weight	IV, Fixed, 95% CI
De Bont 2016	22.9	43	43	17.8	45	45	100.0%	5.10 [-13.29, 23.49]
Total (95% CI)			43			45	100.0%	5.10 [-13.29, 23.49]

Heterogeneity: Not applicable

Test for overall effect: Z = 0.54 (P = 0.59)

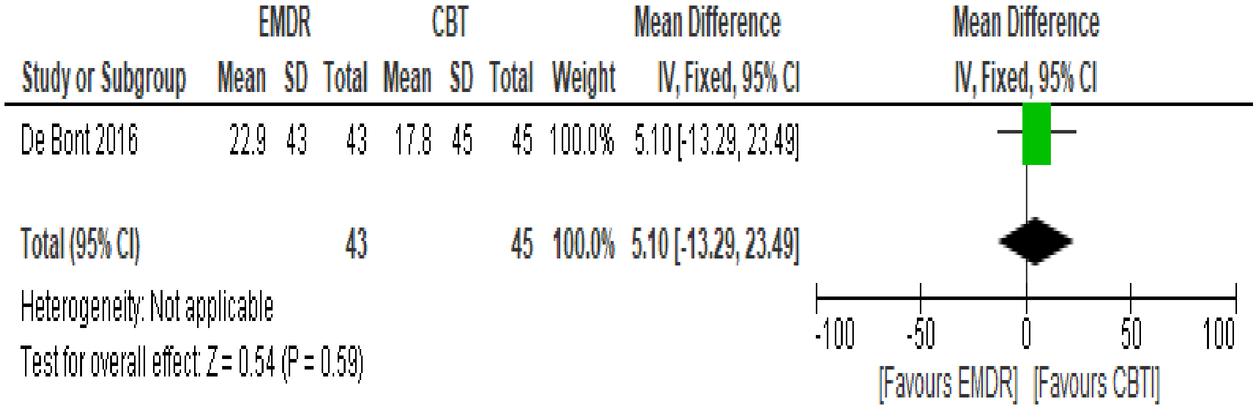

Figure S5: Meta-analysis of the effects of post-treatment on anxiety symptoms

	EMDR			CBT				Mean Difference	Mean Difference
Study or Subgroup	Mean	SD	Total	Mean	SD	Total	Weight	IV, Fixed, 95% CI	IV, Fixed, 95% CI
de Roose 2011	33.3	17.4	26	33.8	18.4	26	2.3%	-0.50 [-10.23, 9.23]	
de Roose 2017	16.53	17.55	43	22.88	21.55	42	3.1%	-6.35 [-14.72, 2.02]	
Diehle 2015	1.6	1.3	25	5.6	3.5	23	94.6%	-4.00 [-5.52, -2.48]	
Total (95% CI)			94			91	100.0%	-3.99 [-5.47, -2.52]	

Heterogeneity: Chi² = 0.80, df = 2 (P = 0.67); I² = 0%

Test for overall effect: Z = 5.30 (P < 0.00001)

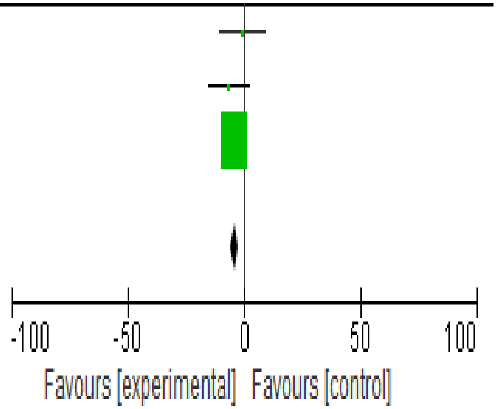

Figure S6: Meta-analysis of the effects of three-month follow-up on anxiety symptoms

	EMDR			CBT				Mean Difference
Study or Subgroup	Mean	SD	Total	Mean	SD	Total	Weight	IV, Fixed, 95% CI
de Roose 2011	33.3	17.4	18	31.6	18.4	20	35.6%	1.70 [-9.69, 13.09]
de Roose 2017	16.53	17.55	42	22.88	21.52	41	64.4%	-6.35 [-14.81, 2.11]
Total (95% CI)			60			61	100.0%	-3.49 [-10.28, 3.30]

Heterogeneity: $Chi^2 = 1.24$, $df = 1$ ($P = 0.27$); $I^2 = 19\%$

Test for overall effect: $Z = 1.01$ ($P = 0.31$)

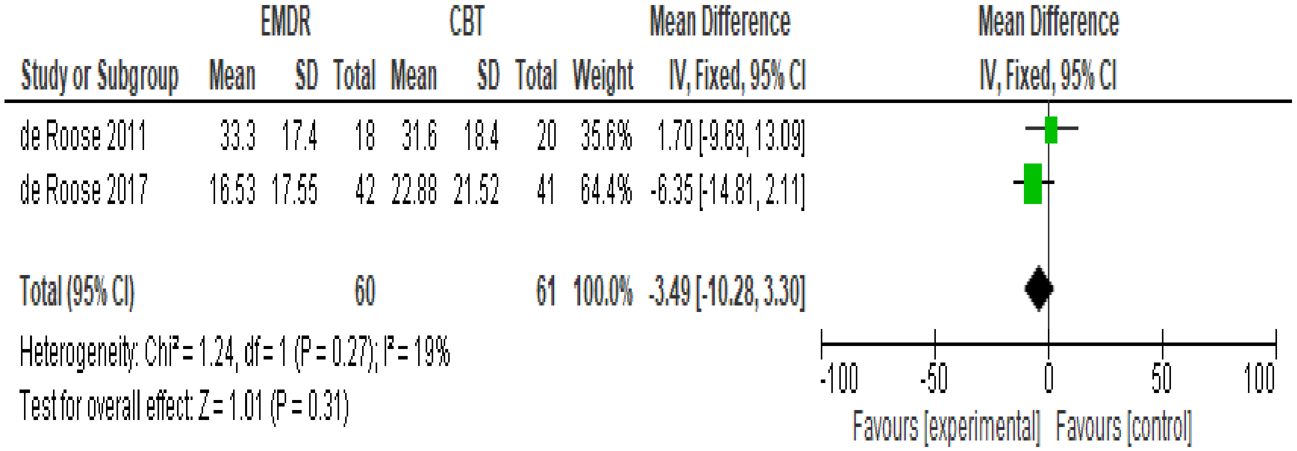

Supplement: Supplementary file 1 [file ijerph-19-16836-s001.zip › ijerph-2062319-supplementary.pdf]
